# Additional Active Movements Are Not Required for Strength Gains in the Untrained during Short-Term Whole-Body Electromyostimulation Training

**DOI:** 10.3390/healthcare11050741

**Published:** 2023-03-03

**Authors:** Holger Stephan, Udo Frank Wehmeier, Tim Förster, Fabian Tomschi, Thomas Hilberg

**Affiliations:** Department of Sports Medicine, University of Wuppertal, Moritzstraße 14, 42117 Wuppertal, Germany

**Keywords:** WB-EMS, electric stimulation, strength training, strength test

## Abstract

Recommendations for conventional strength training are well described, and the volume of research on whole-body electromyostimulation training (WB-EMS) is growing. The aim of the present study was to investigate whether active exercise movements during stimulation have a positive effect on strength gains. A total of 30 inactive subjects (28 completed the study) were randomly allocated into two training groups, the upper body group (UBG) and the lower body group (LBG). In the UBG (*n* = 15; age: 32 (25–36); body mass: 78.3 kg (53.1–114.3 kg)), WB-EMS was accompanied by exercise movements of the upper body and in the LBG (*n* = 13; age: 26 (20–35); body mass: 67.2 kg (47.4–100.3 kg)) by exercise movements of the lower body. Therefore, UBG served as a control when lower body strength was considered, and LBG served as a control when upper body strength was considered. Trunk exercises were performed under the same conditions in both groups. During the 20-min sessions, 12 repetitions were performed per exercise. In both groups, stimulation was performed with 350 μs wide square pulses at 85 Hz in biphasic mode, and stimulation intensity was 6–8 (scale 1–10). Isometric maximum strength was measured before and after the training (6 weeks set; one session/week) on 6 exercises for the upper body and 4 for the lower body. Isometric maximum strength was significantly higher after the EMS training in both groups in most test positions (UBG *p* < 0.001–0.031, r = 0.88–0.56; LBG *p* = 0.001–0.039, r = 0.88–0.57). Only for the left leg extension in the UBG (*p* = 0.100, r = 0.43) and for the biceps curl in the LBG (*p* = 0.221, r = 0.34) no changes were observed. Both groups showed similar absolute strength changes after EMS training. Body mass adjusted strength for the left arm pull increased more in the LBG group (*p* = 0.040, r = 0.39). Based on our results we conclude that concurring exercise movements during a short-term WB-EMS training period have no substantial influence on strength gains. People with health restrictions, beginners with no experience in strength training and people returning to training might be particularly suitable target groups, due to the low training effort. Supposedly, exercise movements become more relevant when initial adaptations to training are exhausted.

## 1. Introduction

Whole-body electromyostimulation (WB-EMS) is a training method that can complement or to some extent replace traditional resistance training, as it can be used alone, superimposed, or combined (different training time points). Since several electrodes are used [1], different muscles can be stimulated at the same time [2]. Strength improvements can be achieved with both high-intensity resistance training and WB-EMS [3]. Previous studies have shown that WB-EMS is applicable in healthy people [4] and also in patients, e.g., in people suffering from Parkinson [5] or sarcopenic obesity [6]. In conventional resistance training, the one repetition maximum (1-RM) is used to describe the training intensity [7]. Since it represents the maximal voluntary contraction, a comparison between electromyostimulation (EMS) and normal contraction is possible [8]. A low-cost way to determine the intensity of strength training is to capture the perceived exertion using the Borg scale [9], which is also used in WB-EMS training [10,11]. In contrast to the 1-RM, where voluntary force production under external load is recorded, the perceived exertion reflects the internal load. For beginners in conventional strength training, at least 2 training sessions per week are recommended. Both multi-joint and single-joint exercises can be performed, using a variety of equipment and the own body weight. Per set, 8 to 12 repetitions should be completed at 60–70% of the repetition maximum [12]. To provide a safe and effective application of WB-EMS, guidelines recommend restricting the duration of one session to a maximum of 20 min. Moreover, the frequency should be limited to one session a week for at least the first eight weeks or a minimum interval of four days should be maintained thereafter [13]. Perceived exertion should be rated approximately as “hard” to “hard+” (lower during initial training) [13], corresponding to 5 to 6 on the Borg CR 10 scale [14]. Nevertheless, in some trials, the training frequencies were higher [2,15], and sometimes lower with one session a week [16,17] compared to the aforementioned recommendation after familiarization. The aggregated training stimulus consists of the number of sessions a week and the length of the training period. Usually, eight sessions or more have been conducted in strength related WB-EMS studies with healthy subjects [10,18]. Early strength improvements due to strength training can be attributed mainly to neural factors. From the third to fifth week on, strength development is mainly caused by hypertrophy [19]. Increases after very few sessions (as seen after three training sessions) are supposedly attributable to lower antagonist activity or motoric improvements of synergists [20]. Elgueta-Cancino and colleagues [21] elicited less inhibitory activity in the cortex, higher corticospinal excitability, and altered motor unit activation as assumed mechanisms of initial strength gain. Muscle growth and strength gain can also be achieved by compact training (eight weeks with three sessions a week) with neuromuscular electrical stimulation [22]. Similar to conventional strength training early strength gains owing to EMS-training are achieved without muscle growth [23].

The body of research on WB-EMS training is growing [24]. EMS can be superimposed on maximum or sub-maximum voluntary dynamic or isometric contractions or applied without any concomitant voluntary contraction. Nevertheless, little is known about the importance of active exercise movements during stimulation. Strength gains due to EMS with exercise movements were previously shown [25,26] and some authors addressed the impact of EMS superimposed on intense strength training [27,28]. To our knowledge, only Kemmler and colleagues [29] investigated the effects of smaller, WB-EMS accompanying movements. In this randomized controlled trial (RCT), participants trained once a week for 12 weeks. However, only older females with little muscle mass were included for the comparison between dynamic use (movements during stimulation) and passive use (only isometric contractions during stimulation) limiting the generalizability of the results obtained. Therefore, the present study aims to investigate whether active exercise movements during stimulation have a positive effect on strength gains of selected upper and lower body muscles in young healthy subjects of both sexes in training sessions using mobile, easily accessible fitness equipment, or the own body mass. We hypothesized that WB-EMS combined with concurrent exercise movements will result in higher strength gains than WB-EMS alone. Hence, this study was designed to clarify whether movement sequences are necessary for strength gains during WB-EMS or, whether the electrostimulation alone induces strength gains. The results might help fitness professionals and EMS-users to optimize recommendations for WB-EMS training depending on individual goals and requirements.

## 2. Materials and Methods

### 2.1. Subjects

The number of subjects to be included in the study was determined using an a priori sample size calculation for statistical comparison of the means of two unpaired groups (using the program GPower 3.1) based on the mean of the effect sizes (Δ strength leg extension: d = 1.67; Δ strength leg flexion: d = 0.79) reported by Kemmler and colleagues [29]. This study is similar to the present study. A predefined lower limit of statistical power of 80% and anα error probability of 0.05 were assumed. A dropout rate of 20% was further added. Based on the results of this calculation, a total of 30 subjects were initially recruited for participation. Subjects were included when being aged between 20 and 40 years and having abstained from physical activity for at least six months prior to the start of the study. Access was possible for both sexes. Subjects were excluded when acute injuries or physical complaints were reported or when contraindications as listed by Kemmler and colleagues [30] or Stöllberger and Finsterer [31] were present (e.g., epilepsy, bleeding disorders). No other exclusion criteria were defined (e.g., BMI, VO_2_max). The study was conducted in accordance with the principles of the Declaration of Helsinki [32] and approved by the ethics committee of the University of Wuppertal (MS/BBL 200114 Wehmeier). All subjects signed a written consent to participate in the study.

### 2.2. Experimental Design

**Figure 1:**
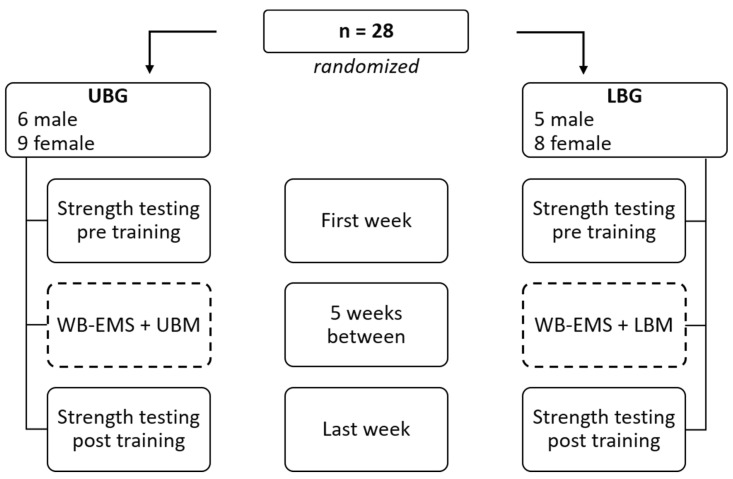
Overview of the study design and procedure. UBG: upper body group; LBG: lower body group; WB-EMS: whole-body electromyostimulation (dashed frame); UBM: upper body exercise movements; LBM: lower body exercise movements.

The procedure was based on a randomized controlled trial design (Figure 1). Subjects were randomly assigned to two training groups (with the program RandList 1.2), with the number of subjects in both groups being equal. In the upper body group (UBG), WB-EMS was accompanied by exercise movements of the upper body only and in the lower body group (LBG) by exercise movements of the lower body only. Therefore, the UBG served as a control when lower body strength is considered, and the LBG served as a control when upper body strength is considered. With this design, WB-EMS without exercises and WB-EMS with exercises could be compared. Intervention duration was set to six weeks, training frequency to one session/week, and the duration of the training session to 20 min. Before and after the training period, maximum force was determined during various exercises. Blinding of subjects was not possible because the intervention is identifiable. Blinding of the investigator was not applicable because the training instructions and the test instructions were given by the same person, a professional EMS trainer with a bachelor’s degree in sports science. Subjects were asked to maintain their dietary habits and to keep their physical activity levels constant, which also meant avoiding additional physical activity. All interventions and measurements were conducted in an EMS studio (go!Orange—Studio für EMS, Remscheid, Germany).

### 2.3. WB-EMS Procedure

Both the UBG and the LBG received the same WB-EMS application (miha bodytec II; miha bodytec GmbH, Gersthofen, Germany) once a week. Subjects wore thin tight-fitting underwear. The vest with wetted electrodes was placed on the upper body and the wetted electrode bands on the arms, buttocks, and legs (miha bodytec). During the 20-min training, the upper and lower back, abdominal muscles, buttocks, muscles around the thigh, chest, and muscles around the upper arm were stimulated with 85 Hz of 350 μs wide rectangular pulses in biphasic mode. Both the duration of the pulse interval (stimulation on) and the pulse pause (stimulation off) were set to 4 s. The pulses were ramped up to the targeted intensity without delay (full intensity directly available) and similarly ramped down to zero (direct interruption of the stimulation) at the end of the stimulation phase. To maintain the same conditions, the stimulation intensity was adjusted to 6–8 on a scale of 1 (hardly noticeable) to 10 (painful) [33]. Regardless of group affiliation, muscles were voluntarily tensed during the stimulation episode.

### 2.4. Exercise Procedure

Both groups received WB-EMS and performed exercises meanwhile. (Supplementary Figures S1–S3). The UBG used upper body exercise movements (chest and upper back including shoulders and arms) and the LBG used lower body exercise movements (buttocks and thigh muscles including abductors and adductors). The UBG training consisted of rowing, butterfly reverse, latissimus pulls, pushups, butterfly, biceps curls, and triceps pulldowns. The LBG training consisted of squats, lunges, adductions, abductions, hip lifts, and leg raises. Both groups exercised the trunk (abdomen and lower back) with back extensions, crunches, and oblique crunches. Selected exercises were performed with additional fitness equipment (fitness tubes and elastic bands, each with varying resistance, and a Swiss Ball). During the first 1 to 2 sessions (depending on the training level), subjects maintained the position over the period of stimulation that they had taken at the onset of the stimulus. One set of 12 repetitions was performed per exercise, with each repetition beginning with the onset of the pulse. To maintain the same physical load level, i.e.,16 to 17 on the Borg RPE scale [33], the number of movements during an impulse interval could be increased up to three. If the training stimulus was not sufficient after the aforementioned customization, the originally targeted static exercise position should be maintained during the interval break. However, overexertion led to a backward correction. Another way to increase the intensity to the desired level was to increase the resistance either by giving an additional fitness tube or rubber band, or by using a version that offered more resistance.

### 2.5. Isometric Strength Testing Procedure

Isometric maximum strength (N) was determined during 10 different exercises (arm adduction, arm pull, leg extension, and leg curl, each unilateral left and unilateral right, as well as during biceps curl and triceps pulldown, each bilateral) in standardized positions (Supplementary Figure S4) pre (initial measurement) and post (final measurement) intervention using a mobile device (KD 9363 including DMS measuring amplifier GVS-2; ME-measuring systems GmbH, Hennigsdorf, Germany), which was more practicable than the determination of the 1-RM. Reliability of the isometric maximum strength measurement method was verified by Runkel and colleagues for several test positions (triceps pulldown, biceps curl, arm pull, sit-up, leg curl, leg extension) in healthy subjects with a comparable body mass index [34] by a high interclass correlation coefficient (r = 0.764 to 0.934). At both time points, the tests were performed three times in each position. The pause was set to 10 seconds between individual tests. In each case, the maximum value was used for analysis. The whole testing procedure lasted approximately 20 min.

### 2.6. Statistical Analysis

Due to the presence of some discordant values (see box plots), skewed distribution in some cases (Shapiro–Wilk test), partial heterogeneity of error variances (Levene’s test), and partial heterogeneity of covariances (Box test), nonparametric statistical tests were employed. The differences between the initial and the final maximum isometric strength were determined separately for each group using the Wilcoxon test. The initial and the final values were compared between the groups using the Mann–Whitney U test. Absolute differences were calculated by subtracting the initial values from the final values, and relative differences were calculated by dividing the final values by the initial values (the initial value was set to 100%). Group comparisons were performed using the Mann–Whitney U test for absolute and relative differences. The significance level was set to < 0.05. Two-tailed analyses were used. The results of the non-parametric tests were used to calculate the effect sizes [35]. A distinction was made between large effects (r ≥ 0.5), medium effects (< 0.5 to 0.3), and small effects (< 0.3 to 0.1) [36]. Statistics were calculated using SPSS (IBM SPSS Statistics for Windows, Version 28.0., IBM Corp., Armonk, NY, USA) and Excel (Microsoft Excel for Windows, 16.0., Microsoft Corp., Redmond, WA, USA). An intention-to-treat analysis was not possible due to dropouts occurring at baseline.

## 3. Results

**Figure 2:**
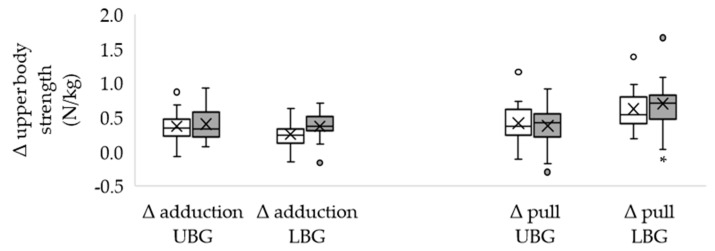
Differences between the final and the initial body weight adjusted maximum upper body strength (arm adduction and arm pull) in the upper body group and in the lower body group. Δ: differences; UBG (*n* = 15): upper body group; LBG (*n* = 13): lower body group; white box: right arm; grey box: left arm; circles represent discordant values; means are displayed by crosses and medians by crossbars; * significant difference between LBG and UBG, *p* < 0.040 (effect size r = 0.39).

**Figure 3:**
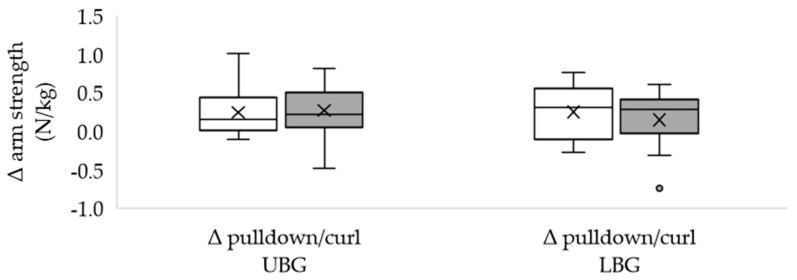
Differences between the final and the initial body weight adjusted maximum arm strength (biceps curl and triceps pulldown) in the upper body group and in the lower body group. Δ: differences; UBG (*n* = 15): upper body group; LBG (*n* = 13): lower body group; white box: triceps pulldown; grey box: biceps curl; the circle represents a discordant value; means are displayed by crosses and medians by crossbars; no significant differences occurred.

**Figure 4:**
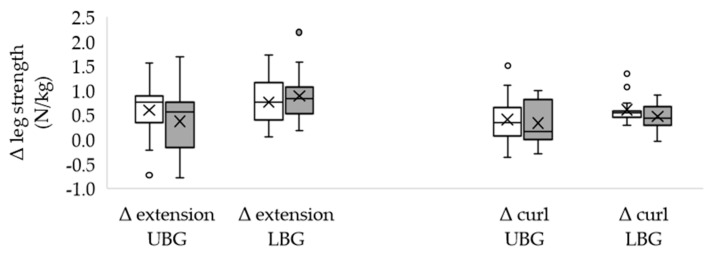
Differences between the final and the initial body weight adjusted maximum leg strength (leg extension and leg curl) in the upper body group and in the lower body group. Δ: difference; UBG (*n* = 15): upper body group; LBG (*n* = 13): lower body group; white box: right leg; grey box: left leg; circles represent discordant values; means are displayed by crosses and medians by crossbars; no significant differences occurred.

Of the included subjects, 28 completed the study. The dropouts occurred due to personal reasons. The characteristics of the groups did not differ significantly from each other (Table 1) and the total training volume was similar in both groups. Most subjects (*n* = 9 in each group) completed five sessions and no adverse effects occurred. The body mass remained unchanged in both the UBG and the LBG (Table 1). Neither the initial nor the final values differed significantly between the two groups. Isometric maximum strength was significantly higher after EMS training in both groups, both in absolute terms (Table 2 UBG; Table 3 LBG) and body mass adjusted (N/kg), except for left leg extension in the UBG and biceps curl in the LBG. The changes in absolute strength were similar in both groups (Table 4). Body mass adjusted strength during left arm pull showed a higher increase in the LBG (Figure 2). In the other test positions, group affiliation made no difference (Figure 2, Figure 3 and Figure 4). Furthermore, the LBG achieved a higher percentage strength gain in left arm pull, both absolute (Table 4) and body mass adjusted (UBG median 114.25% vs. LBG median 137.05%; *p* = 0.020; r = 0.44).

## 4. Discussion

### 4.1. Overview

Significant strength changes were observed in both groups after about five weeks training (one session per week). The percentage differences between the initial and final tests were higher than those found in the reliability analysis of the test device by Runkel and colleagues [34]. Therefore, the changes could be attributed to training. LBG training improved left arm pull strength more than UBG training. However, there were no group differences in the other exercises. Initial values between the two groups were not significantly different, but possibly at clinically relevant levels. If the higher initial values had been due to differences in training history, a lower ability to further increase strength would have be needed to be considered [37]. However, subjects should have abstained from intense physical activity for at least six months before starting the study.

### 4.2. Accompanying Voluntary Activity

Little is known about the effects of movements for strength gain during EMS. During local application, movements are usually avoided and isometric contractions are performed. Maffiuletti [38] summarized that there are no differences in strength increase between EMS and EMS superimposed on voluntary contractions. However, the conclusion is based on the results of isometric interventions. Although movements are thought to promote the activity of stimulated muscles [26], our results failed to show a consistent influence of active exercise movements on strength gains. Furthermore, strength gains from conventional resistance training depend, among others, on the range of motion used [39,40]. However, isometric contractions at multiple joint angles might cover at least in part the physiological range of motion. For EMS training, Maffiuletti [38] recommends changing the joint position and furthermore, changing the electrode positioning to increase recruitment. Admittedly, Kemmler and colleagues [29] demonstrated the benefit of movement during WB-EMS use, with participants exercising in supine position. In contrast, our participants performed exercises in different positions. Therefore, any movements of body parts that were not primarily intended for the exercises and possible differences in resistance to gravity might have influenced the results. Furthermore, it needs to be considered that additional fitness equipment (fitness tubes and elastic bands with different resistance as well as a Swiss Ball) was used for selected exercises. However, exercise movements using additional fitness equipment did not affect the results. In addition, both the UBG and LBG performed exercises for the trunk. Therefore, both groups received partially similar dynamic training stimuli (three exercises). Movements inevitably lead to changes in muscle length and shape (e.g., biceps muscle during curl). Hence, changes in the electrode contact were very likely to occur. Furthermore, training that aims to enhance endurance and strength at the same time, such as EMS superimposed on cycling [41,42], requires movements. However, stimulation intensity must be considered to ensure the range of motion [43].

### 4.3. Training Models and Adaptations

Supraspinal mechanisms appear to be responsible for the initial strength development through EMS training [23]. Bezerra and colleagues [44] showed increased strength after EMS superimposed onto maximum isometric quadriceps contractions, not only of the exercised leg but also of the unexercised leg, confirming neural contribution. The potential to use EMS for rapid strength gains was demonstrated by Deley and colleagues [45], who reported that maximum dynamic leg extension torque in prepubertal girls could be increased by up to 50.6% with three weekly isometric applications over a three-week period. According to Adams [46], atrophic patients as well as casualties are target groups for the use of EMS. After 5 to 6 weeks, a 10 to 15% enhancement of muscle function can be achieved, but three sessions a week are recommended. Several studies confirmed the impact of WB-EMS on strength [10,26]. However, to our knowledge, only Kemmler and colleagues [29] have studied the effects of exercise during WB-EMS to date. In most cases, the lower body was investigated. Von Stengel and Kemmler [25] showed that leg/hip strength can be improved with 1.5 WB-EMS training sessions (with unloaded, low effort exercises) per week over a 14 to 16 week period, regardless of age. Furthermore, strength gains due to unloaded WB-EMS were similar compared to a HIT training after 16 weeks with three sessions in two weeks [3]. An increase in strength was also observed after shorter training periods. For example, WB-EMS superimposed on jumps twice a week over seven weeks significantly improved leg strength in contrast to normal jump training [10,47,48]. In the study by Wirtz and colleagues [28], leg flexors strength increased only after combining stimulation of multiple body parts with loaded squats (100% 10 RM) twice per week and it was higher three weeks after the six-week training compared to the same training without stimulation. Dörmann and colleagues [18] showed significant improvements in leg strength after a four-week, eight-session WB-EMS training program that were similar to those seen in the control group, which performed the same training that included strength exercises, without additional stimulation, and in which intensification was accomplished using other training tools. However, not only leg muscles but also upper body muscles could benefit from dynamic WB-EMS. Reljic and colleagues [26] observed improvements throughout the entire body after a 12-week WB-EMS program with slight motions, consisting of two sessions per week. Our results suggest that even fewer training sessions are beneficial than previously described, whether or not exercise movements are performed during stimulation, which appears to be due to neural factors. Therefore, not only locally applied EMS training regimens have the potential to increase strength, but also WB-EMS training regimens without additional exercise movements.

### 4.4. Transferability

Benefits from WB-EMS can also be expected, for example, for patients suffering from sarcopenia, sarcopenic obesity, and low back pain [14]. It might be useful especially for beginners to start WB-EMS training with a five-week training period without additional exercise movements to improve basic strength before starting a more challenging exercise program. WB-EMS without additional exercise movements can be a first access to training when health conditions do not allow conventional exercises or when a lack of compliance exists. Relative to WB-EMS, local application appears to be superior in gaining strength [49]. However, the lack of focus on selected zones owing to stimulation of the entire body is a suggested explanation for the difference [14]. Therefore, only target muscles could be stimulated and not all available electrodes could be used, even if an electrode suit is worn, or zones could be stimulated in an individual order.

### 4.5. Limitations

We have shown that the effect of WB-EMS on strength gains is independent of the concomitant exercise movements. Nevertheless, some limitations need to be acknowledged. A test of core strength would have been useful, as both groups performed core strength exercises under the same conditions and a higher strength can be expected as observed in the study by Berger and colleagues [1], although they used a more extensive training program. Owing to two dropouts, the group sizes were slightly different, which affected the comparison. Furthermore, the strength gains of the dynamically trained muscles might have been underestimated, since only isometric strength was tested. It must also be mentioned that the increase in strength might have been influenced by deviations from the predefined number of training sessions. To evaluate the intensity of the movement sequences, an unstimulated group could have been used. Furthermore, an inactive group could have been used as a reference for the interventions. However, the study focused on the comparison between the EMS application without and the application with concurring exercise movements. When using WB-EMS training technology, the load parameters must be set with care to avoid unintended side effects, particularly during the first sessions of novices when adaptation to the load has not yet occurred in the form of the “repeated bout effect” [50].

## 5. Conclusions

WB-EMS training without accompanying movement exercises leads to substantial strength gains even during a short WB-EMS training period. At the beginning of WB-EMS training, electromyostimulation is more important for strength gains than active exercise movements. Therefore, future studies should examine the effects of exercise movements during long-term training periods, or consider individuals already adapted to WB-EMS training or strength training. The transferability of the results to a collective experienced with WB-EMS or strength training should be questioned, as movements (and maybe other approaches, e.g., additional mass or complicating tasks) may become more relevant when initial adaptations to training are exhausted. Since the training effort with WB-EMS is low, people with health restrictions, beginners without experience in strength training, and those returning to training might benefit from these results. These groups could refrain from exercise movements during the first WB-EMS training sessions and integrate them during the course of the subsequent training.

## Figures and Tables

**Table 1:** Characteristics presented as medians (ranges) of the total collective (*n* = 28) and the two groups.

	Total (m 11; f 17)	UBG (m 6; f 9)	LBG (m 5; f 8)
Age [years]	28 (20–36)	32 (25–36)	26 (20–35)
Height [cm]	173.0 (159–186)	174.0 (159.0–186.0)	171.0 (160.0–186.0)
Body mass pre [kg]	74.1 (47.4–114.3)	78.3 (53.1–114.3)	67.2 (47.4–100.3)
Body mass post [kg]	74.4 (48.0–112.9)	78.2 (52.8–112.9)	68.0 (48.0–99.7)
BMI pre [kg/m^2^]	25.33 (18.21–40.98)	25.68 (19.27–40.98)	23.88 (18.21–30.35)
BMI post [kg/m^2^]	25.06 (18.08–38.57)	25.63 (19.16–38.57)	23.74 (18.08–30.65)
Number of sessions	5 (3–6)	5 (3–6)	5 (3–6)

m: male; f: female; UBG: upper body group; LBG: lower body group; BMI: body mass index.

**Table 2:** Initial and final median maximum strength values (ranges) of the upper body group (UBG).

Test Position	Strength (N) Initial	Strength (N) Final	Significance	Effect Size r
Arm adduction right	83.3 (44.8–143.0)	116.2 (42.6–178.3)	0.002 **	0.81
Arm adduction left	83.0 (44.8–124.4)	124.4 (54.2–196.0)	<0.001 **	0.88
Arm pull right	173.0 (117.0–293.7)	232.6 (124.7–331.6)	0.001 **	0.84
Arm pull left	196.0 (114.1–331.7)	242.2 (132.4–378.3)	0.006 **	0.70
Triceps pulldown	253.0 (142.7–461.2)	279.2 (149.6–510.4)	0.012 *	0.65
Biceps curl	308.5 (117.6–512.7)	331.7 (143.6–528.2)	0.008 **	0.69
Leg extension right	377.2 (196.7–697.4)	404.8 (237.4–766.0)	0.015 *	0.63
Leg extension left	373.4 (130.9–682.5)	403.3 (218.6–769.0)	0.100	0.43
Leg curl right	184.6 (40.9–296.1)	200.1 (71.2–447.5)	0.005 **	0.72
Leg curl left	185.5 (48.0–296.5)	186.9 (85.5–396.0)	0.031 *	0.56

*n* = 15; * significant difference *p* < 0.05; ** highly significant difference *p* < 0.01.

**Table 3:** Initial and final median maximum strength values (ranges) of the lower body group (LBG).

Test Position	Strength (N) Initial	Strength (N) Final	Significance	Effect Size r
Arm adduction right	61.7 (28.8–127.4)	84.9 (43.1–155.5)	0.007 **	0.75
Arm adduction left	56.2 (29.0–111.3)	85.1 (46.1–170.0)	0.002 **	0.84
Arm pull right	140.0 (80.9–281.7)	170.4 (145.0–362.2)	0.001 **	0.88
Arm pull left	131.0 (94.9–216.8)	167.9 (136.8–378.2)	0.001 **	0.88
Triceps pulldown	178.0 (125.0–474.0)	203.0 (140.2–474.2)	0.039 *	0.57
Biceps curl	215.3 (137.0–559.5)	212.7 (167.9–531.0)	0.221	0.34
Leg extension right	330.5 (218.2–725.0)	385.6 (281.0–787.8)	0.002 **	0.86
Leg extension left	304.0 (184.8–612.0)	348.5 (228.1–704.9)	0.001 **	0.88
Leg curl right	139.4 (104.6–268.7)	170.0 (140.0–311.9)	0.001 **	0.88
Leg curl left	127.0 (105.0–261.4)	166.2 (132.0–287.9)	0.002 **	0.86

*n* = 13; * significant difference *p* < 0.05; ** highly significant difference *p* < 0.01.

**Table 4:** Median differences (ranges) between final and initial maximum strength values in both groups (UBG and LBG).

Test Position	Δ UBG (N)	Δ UBG (%)	Δ LBG (N)	Δ LBG (%)
Arm adduction right	26.5 (−11.9–68.3)	137.01 (83.43–175.63)	12.6 (−13.9–44.3)	120.68 (86.47–222.92)
Arm adduction left	27.5 (5.6–83.3)	131.70 (104.61–183.05)	29.1 (−15.2–58.7)	152.74 (84.85–244.67)
Arm pull right	29.4 (−6.3–85.4)	114.74 (96.14–149.36)	32.5 (10.4–96.1)	128.46 (105.28–182.82)
Arm pull left	21.6 (−27.2–90.2)	115.07 (84.68–144.24)	41.4 (4.6–164.0)	131.67 (102.47–176.56) *
Triceps pulldown	11.7 (−9.0–89.5)	105.88 (97.20–161.89)	19.6 (−22.5–74.5)	110.17 (93.72–130.56)
Biceps curl	24.5 (−31.3–69.4)	107.45 (88.79–139.86)	21.2 (−45.1–40.7)	108.82 (88.02–122.55)
Leg extension right	52.0 (−90.7–141.2)	117.40 (86.99–125.25)	55.1 (−4.4–130.3)	116.67 (98.94–135.90)
Leg extension left	41.8 (−94.5–120.3)	110.05 (80.11–167.00)	56.0 (4.7–215.7)	121.68 (101.14–144.09)
Leg curl right	30.3 (−27.8–151.4)	117.45 (79.41–185.79)	37.1 (15.5–126.3)	124.87 (111.12–168.34)
Leg curl left	13.9 (−27.3–99.5)	107.42 (88.28–178.13)	39.7 (−1.7–70.7)	131.41 (98.79–160.84)

Δ: differences; UBG (*n* = 15): upper body group; LBG (*n* = 13): lower body group; * significant difference compared to UBG, *p* = 0.020 (effect size r = 0.44).

## Data Availability

Data are available (Table S1).

## Supplementary Materials

The following supporting information can be downloaded at: https://www.mdpi.com/article/10.3390/healthcare11050741/s1, Figure S1: Exercise movements for the upper body performed by the upper body group (UBG); Figure S2: Exercise movements for the lower body performed by the lower body group (LBG); Figure S3: Exercises for the trunk performed by both groups; Figure S4: Isometric strength tests; Table S1: Data overview.
