# Supplementary material for: Additional Active Movements Are Not Required for Strength Gains in the Untrained during Short-Term Whole-Body Electromyostimulation Training
Source: Healthcare (Basel). 2023 Mar 3;11(5):741. doi: 10.3390/healthcare11050741 (PMC10000632; doi:10.3390/healthcare11050741)
Supplement: Supplementary file 1 [file healthcare-11-00741-s001.zip › supplement Figures S1-S4.pdf]

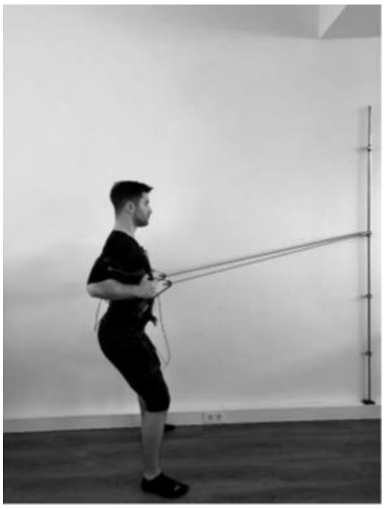

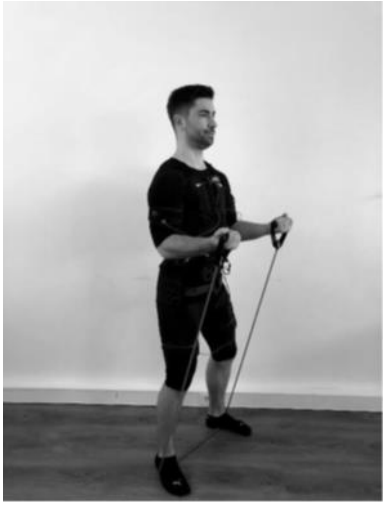

a b

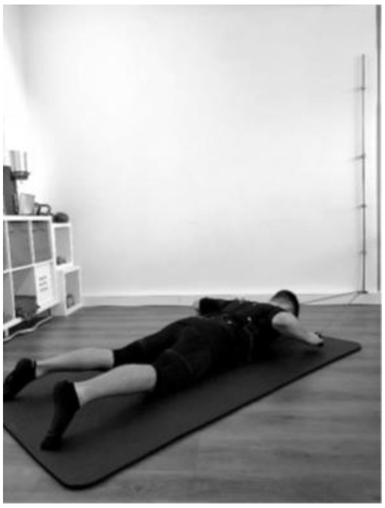

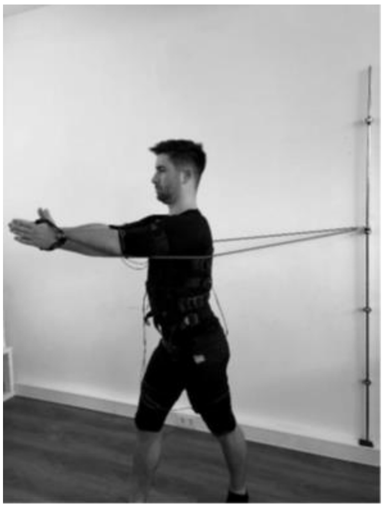

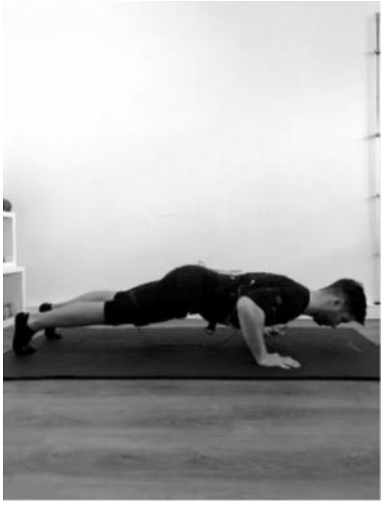

c d e

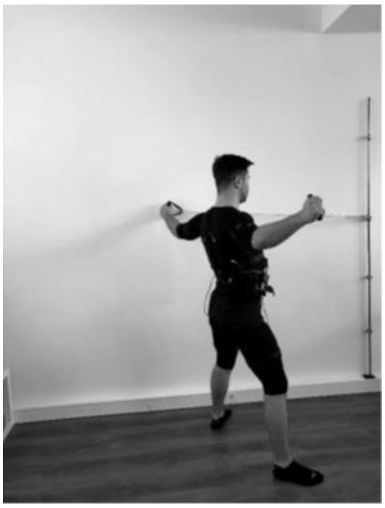

f g

Figure S1. Exercise movements for the upper body performed by the upper body group (UBG). a: rowing (pull arms bent backwards); b: biceps curls (bend of the supinated elbows fixed to the body); c: latissimus pulls (pull arms down bent); d: butterfly (raise arms to shoulder height and pull them forward in an extended position); e: pushups (push up, hands on height of breast, arms somewhat spread apart); f: butterfly reverse (raise arms to shoulder height and pull them backwards almost stretched; g: triceps pulldowns (extension of the supinated elbows fixed to the body)

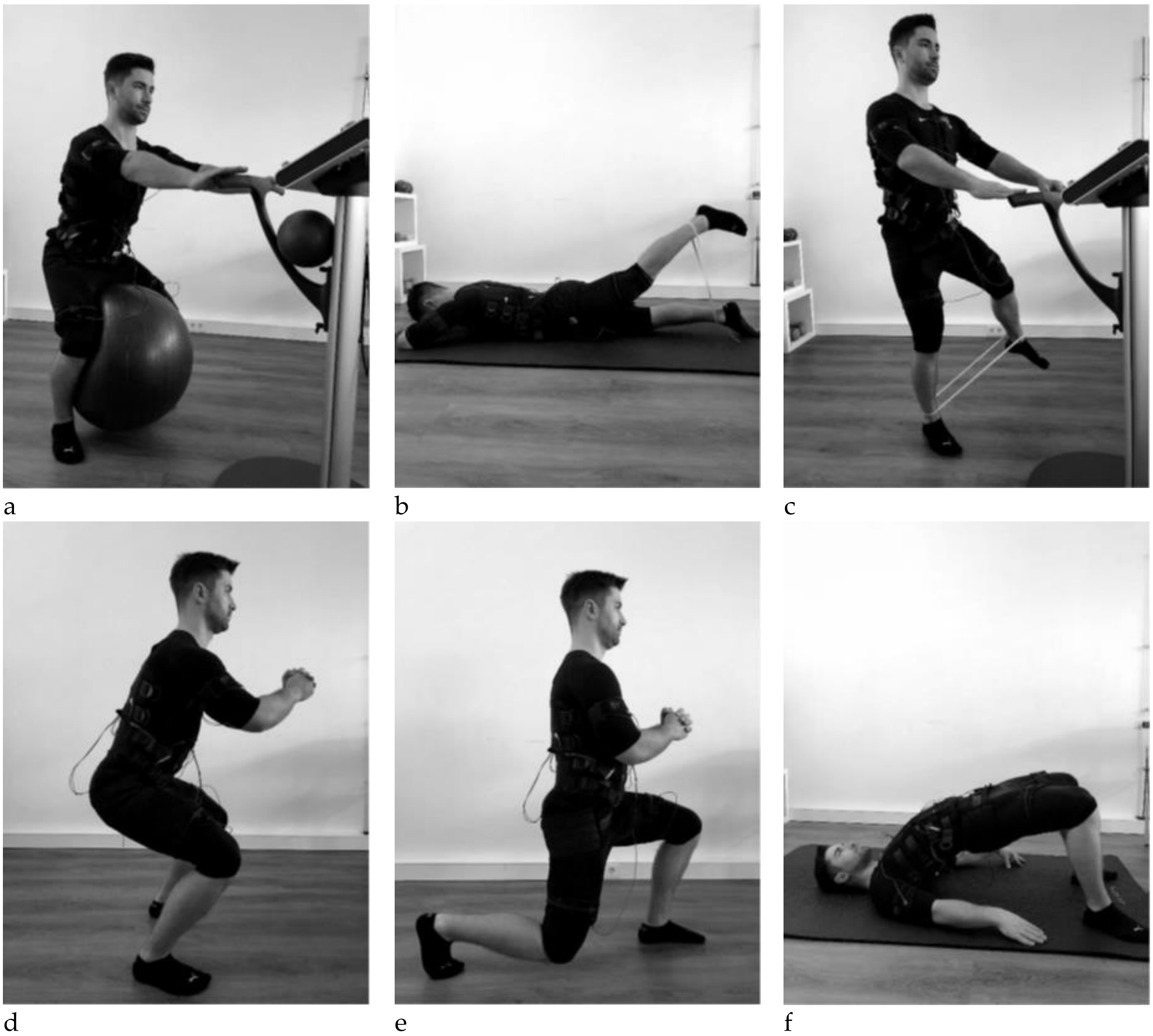

Figure S2. Exercise movements for the lower body performed by the lower body group (LBG). a: adductions (squeeze Swiss Ball with bent knee joints); b: leg raises left and right (lift stretched leg); c: abductions left and right (lift stretched leg sideways); d: squats (take a sitting position without a chair); e: lunges left and right (in step position weight on the front leg, 90 degrees bend both knee joints, do not put down the back knee, do not move the front knee beyond the tip of the foot); f: hip lifts (raise the buttocks and straighten the hips)

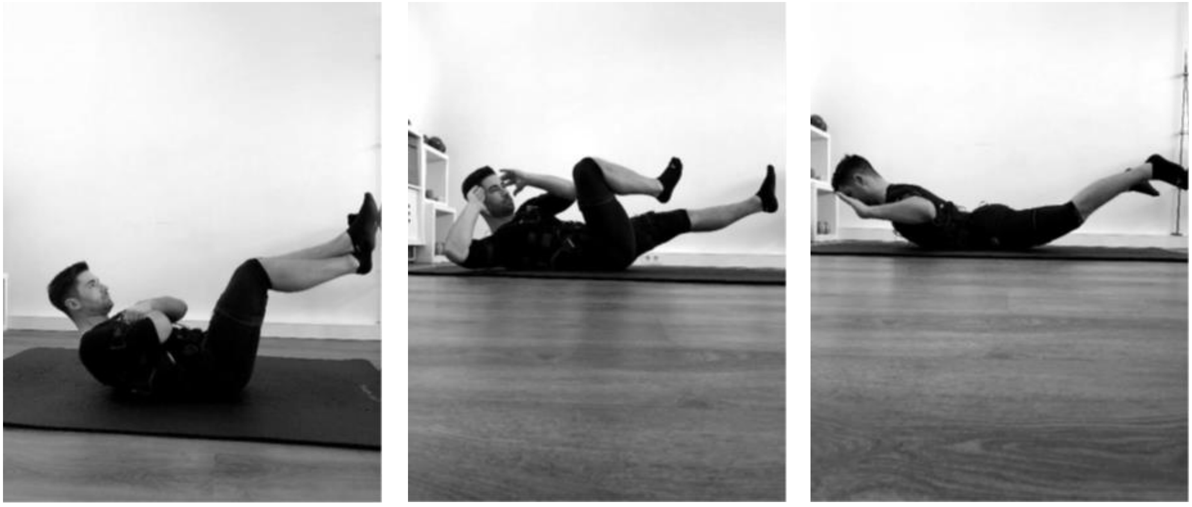

a b c

Figure S3. Exercises for the trunk performed by both groups.
a: crunches (raise the upper body with the knee joints bent 90 degrees and the hip joints bent 90 degrees); b: oblique crunches (alternately straighten one leg and bend one leg, lifting the upper body and pulling the opposite elbow to the bending knee); c: back extensions (raise stretched legs, head, and bent arms)

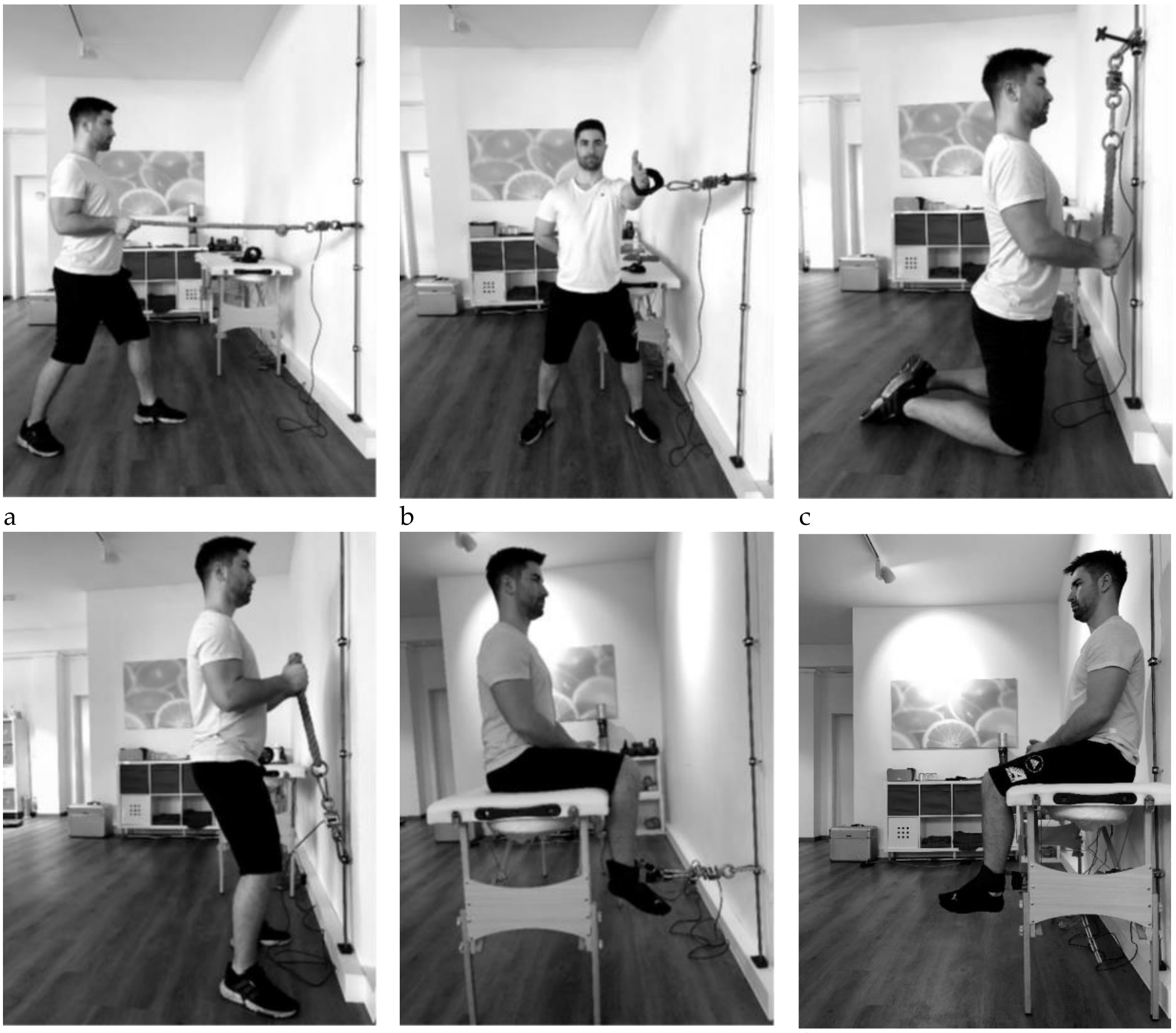

a b c

d e f

Figure S4. Isometric strength tests.
a: arm pull (left and right); b: arm adduction (left and right); c: triceps pulldown; d: biceps curl; e: leg curl (left and right); f: leg extension (left and right)
